# Dental anxiety in general dental practitioner’s everyday life

**DOI:** 10.3389/fdmed.2026.1987579

**Published:** 2026-10-23

**Authors:** Tiril Willumsen, Charlotte Knutsen, Mathilde M. Gran, Anne Rønneberg

**Affiliations:** Department of Paediatric Dentistry and Oral Health Psychology, Institute of Clinical Dentistry, Faculty of Dentistry, University of Oslo, Oslo, Norway

**Keywords:** avoidance, dental anxiety, dental pain, local anaesthesia, preventive care

## Abstract

**Introduction:**

Dental anxiety is a significant contributor to poor oral health, and pain plays a central role in its development. Adequate use of local anesthetic injections is therefore essential for preventing and reducing anxiety in dental settings. This study aimed to estimate how often Norwegian dentists encounter patients with dental anxiety in routine clinical practice and to examine the association between anticipatory dental anxiety and anxiety related to injections of local anesthetic.

**Methods:**

A total of 5,880 dental patients from all regions of Norway completed the Modified Dental Anxiety Scale (MDAS).

**Results:**

General dental practitioners (GDPs) in Norway were found to encounter a substantial proportion of patients with clinically relevant dental anxiety: approximately one in ten patients reported moderate dental anxiety (MDAS ≥ 15), and one in twenty met the threshold for high dental anxiety (MDAS ≥ 19). The highest anxiety scores were observed among women and patients aged 20‣40 years. Among patients with high anticipatory anxiety, eight out of ten also reported clinically significant fear of injections.

**Discussion:**

These findings underscore the need for systematic assessment and early management of dental anxiety, including both anticipatory anxiety and injection-related anxiety, to safeguard oral health and ensure ethically sound, high-quality dental care.

## Introduction

Dental anxiety is an important contributor to the development of poor oral health, and, to quote the World Health Organization, «No health without oral health.» (1).

In the 1980s, Berggren introduced the “vicious circle” model, describing how anxiety leads to avoidance of dental care, deterioration of oral health, and subsequent feelings of shame and guilt (2). Numerous scientific publications have supported this model, which has since been further elaborated into a spiral to reflect the progressive nature of the condition (3–7).

Anxiety typically develops gradually, and consequently general dental practitioner (GDPs) are essential in preventing, early intervention and treatment of dental anxiety. Individuals who enter dental situations with degrees of anticipatory anxiety may, if their anxiety is not adequately addressed, perceive treatment as unpleasant or painful. Over time, this may lead to avoidance behavior and progression into the vicious circle. Clinically, patients with dental anxiety often postpone routine care and seek treatment only when experiencing acute pain, while necessary rehabilitative procedures remain uncompleted.

When patients’ fear and anxiety are addressed appropriately, anxiety may be reduced (5, 8, 9). Given that effective anxiety management can reduce the likelihood of oral disease, it may be argued that it should form an integral part of GPD's everyday practice and be addressed alongside other preventive measures, such as dietary counselling and oral hygiene instruction.

Pain consistently plays a central role in the development of dental anxiety. Nermo et al. (10) examined adolescents aged 15–18 years and found that approximately 50% experienced changes in dental anxiety over time. Among those with treatment experience, anxiety decreased in individuals who reported no pain, whereas it increased among those who had experienced pain. As adequate local anaesthesia is fundamental to pain-free dental treatment, avoidance of injections due to fear may contribute to the maintenance and progression of dental anxiety. Vika et al. (11) highlighted a significant overlap between blood-injection-injury phobia and dental anxiety, and Berge et al. (12) found that intraoral injections are among the most anxiety-inducing stimuli in the dental treatment situation.

Pain and anxiety affect each other mutually in a negative interaction. Previously painful treatments at the dentist as a child are known to be a major risk factor for developing dental anxiety (5, 13–16) while high anxiety is also one of the most important predictors of increased pain perception during treatment (17).

In a clinical context it may be difficult to determine whether a patients’ refusal of local anaesthetics represent an appropriate decision or is instead a consequence of avoidance of an injection due to anxiety. In this context, it is of interest to explore the extent to which high anticipatory anxiety reflects underlying injection anxiety, as evidence on this association remains limited.

There is strong evidence supporting structured treatment approaches for dental anxiety in specialist clinics, particularly cognitive behavioral therapy (CBT) delivered by interdisciplinary teams of psychologists and dentists (9). In contrast, dental anxiety management in general practice has received less attention, although the limited number of available studies report promising outcomes (18–20).

Despite this, the diagnosis and management of dental anxiety remain limited in general dental practice. Humphris et al. (21) have emphasized the importance of routinely incorporating dental anxiety assessment. However, clinical experience suggests that few dentists routinely assess anxiety during anamnesis, and reimbursement systems rarely include fees for anxiety management. For anxiety treatment to be implemented effectively, costs must be clarified and appropriate funding mechanisms established. Barriers may include uncertainty among dentists regarding professional responsibility, lack of confidence in delivering such treatment, and patient-related factors such as low motivation or limited access to care.

To estimate the need for anxiety management in general practice, it is necessary to understand how frequently GDPs encounter patients with dental anxiety in everyday practice. Although epidemiological studies provide prevalence estimates, these likely underestimate the true burden, as individuals with high anxiety often do not participate in population surveys. Prevalence estimates based on specialist clinic populations also exclude individuals who, for various reasons, do not seek specialist care. Consequently, dental anxiety among regular dental patients has not received sufficient attention (22).

The aims of this study are therefore:

(i) to determine proportion and age distribution of dental anxiety among patients attending routine dental care in Norway, and
(ii) to explore the association between anticipatory dental anxiety and anxiety related to injections of local anesthetic

Two hypotheses were developed:

1. Dental practitioners (GDPs) in Norway encounter a substantial proportion of patients with dental anxiety
2. Patients who report high dental anxiety the day before and in the waiting room before dental treatment have high risk of being anxious for injections of local anesthetics.

## Materials and methods

### Study design and setting

Between 2015 and 2022, twelve courses were conducted within the Norwegian Dental Association's Continuing Education Programme (TSE), Module 8: Special Clinical Challenges. Each course comprised two “two-day sessions” held approximately two months apart, with mandatory assignments in the interim period. A total of 442 dentists and 12 dental hygienists participated, representing all regions of Norway and both public and private dental services.

### Data collection

As part of the course assignment, the participants were instructed to distribute a paper-based questionnaire to all patients attending scheduled appointments during one week of choice between the two course sessions. Dental assistants handed out the questionnaires in the waiting room prior to the appointment to allow sufficient time for completion. Completed questionnaires were returned to the main researchers (first author) by post, who entered the anonymised data into SPSS.

### Inclusion criteria

- Patients aged ≥12 years
- Attendance at a scheduled appointment with a dentist or dental hygienist during the designated week in the interim period

### Exclusion criteria

- Insufficient Norwegian language proficiency to read and understand the questionnaire

### Questionnaire

#### Background variables

Participants reported age, gender, and county of residence.

#### Dental anxiety measure

Dental anxiety was assessed using the Modified Dental Anxiety Scale (MDAS).

The Modified dental anxiety scale, MDAS, was introduced in 1995 by Gerald Humphris. It was a further development of the well-established dental anxiety measure DAS (23, 24), by adding a fifth question dealing with the fear of injections and local anesthesia.

MDAS has currently been translated into 30 languages (25) and is today a validated questionnaire that has been used in research, but also as a tool for dentists who want to survey anxious patients in clinics. The form has also been validated for use in Norwegian (26).

MDAS consists of five items, each item is assessed with a Lickert scale: 1 = not anxious, 2 = slightly anxious, 3 = fairly anxious, 4 = very anxious, 5 = extremely anxious. The total MDAS score range 5–25. MDAS scores were dichotomised using thresholds >14 (moderate dental anxiety) and >18 (high dental anxiety) (27).

After using confirmatory factor analysis, proposed a two-factor structure—anticipatory anxiety and treatment-related anxiety—with reliability coefficients of 0.74 and 0.86, respectively (28).

To assess injection-related anxiety, question 5 of the MDAS was analysed separately. A new variable was created to identify respondents scoring >2, >3, and exactly 5 on this item.

To explore a clinically relevant situation—namely, whether GDPs can infer a patient's fear of injections on the basis of anticipatory anxiety (MDAS 1 and MDAS 2)—we examined this factor in relation to the patient's reported fear of injections (MDAS 5)

#### Data management and ethical considerations

Questionnaires were distributed and collected locally before being forwarded to the first author. As no information on the number of questionnaires distributed or the number of clinicians and patients at each site was available for the research team, response rates could not be calculated.

All questionnaires were completed anonymous. Neither clinicians nor patients were directly or indirectly identifiable after submission. Consequently, the study did not involve the processing of personal data and was not subject to notification to Regional Ethics Committee (REC) or Norwegian Agency for Shared Services in Education and Research (Sikt).

### Statistical analysis

Data were analysed using IBM SPSS Statistics 30. Descriptive statistics were generated for all variables. Chi-square tests were used for non-parametric comparisons.

Although MDAS scores were not normally distributed, both mean and standard deviation were reported, consistent with previous literature.

As all data were collected anonymously on paper without identification of patients or dentists, no ethical approvals were relevant.

## Results

A total of 5,880 completed MDAS questionnaires were returned. Of these respondents, 49.2% identified as female and 42.4% as male, while 8.4% did not report gender. When excluding missing data, the gender distribution corresponded to 53.6% females and 46.3% males.

Participants ranged in age from 12 to 93 years. The largest age group was adolescents aged 12–18 years (*n* = 1,183; 22.1%). The mean age of the sample was 41.4 years (SD = 21.6).

Overall, the mean MDAS score for the sample was 9.1 (SD = 4.3). Using established thresholds, 12.0% of respondents scored in the range defined as moderate dental anxiety (MDAS ≥ 15) and 5.3% scored in the range defined as high dental anxiety (MDAS ≥ 19).

Gender differences. Mean MDAS score was statistically significantly higher among females (9.8, SD = 4.62) than males (8.27, SD = 3.84), *p* < 0.01. A chi-square test indicated that a significantly greater proportion of females reported elevated anxiety compared with males: MDAS ≥ 15: 15.7% (females) vs. 7.9% (males); MDAS ≥ 19: 7.2% (females) vs. 3.0% (males); difference significant at *p* < 0.01.

Figure 1 illustrates the percentage of participants within each age category with MDAS scores ≥ 15 and ≥ 19. The prevalence of elevated MDAS scores varied by age group. The highest proportion with MDAS ≥ 15 was observed in the 30–39 years age band (23%), followed by 19–29 years (18%). Lower prevalence was observed in older age groups.

**Figure 1:**
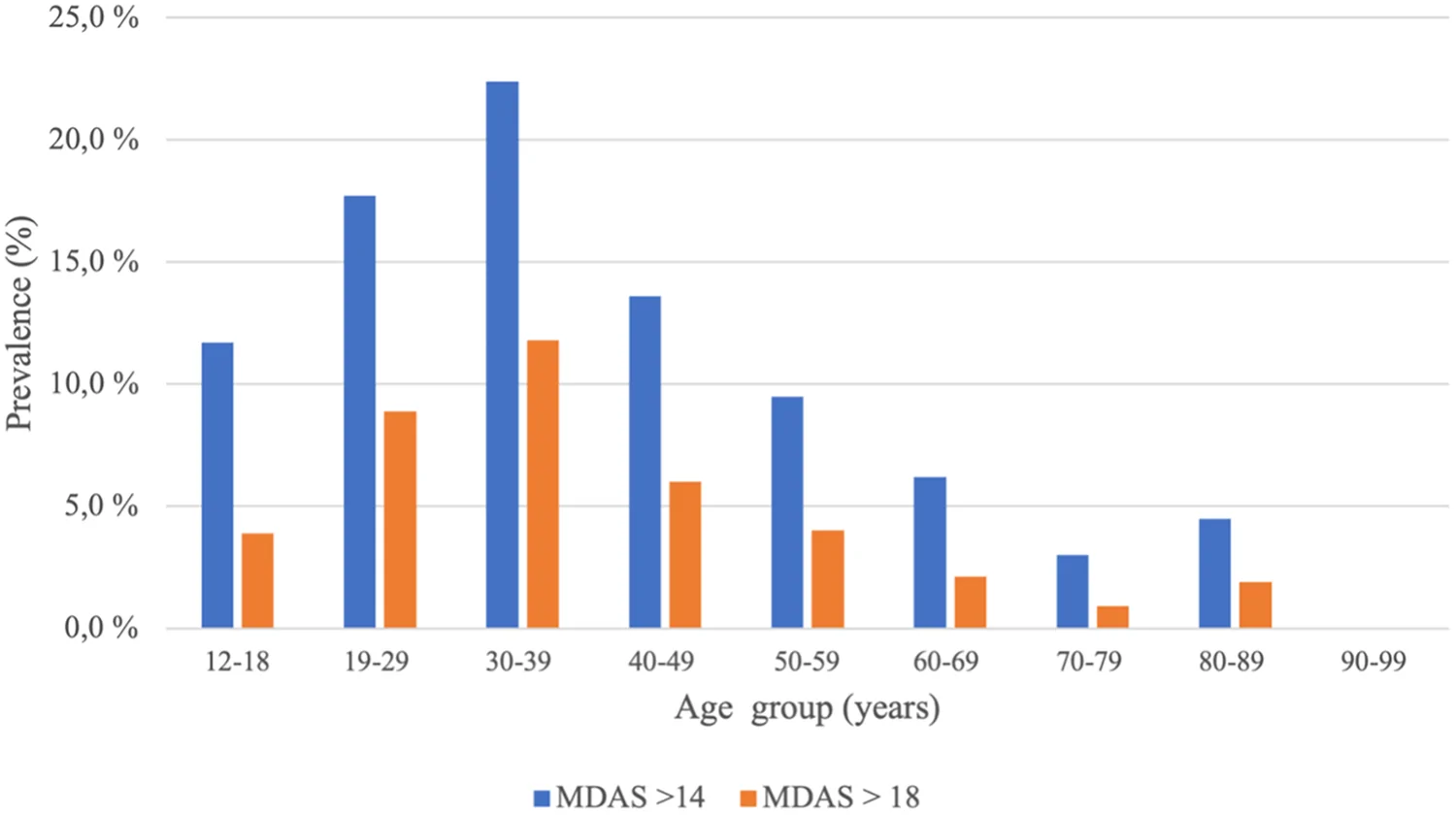
Illustates two levels of MDAS in different age groups.

The mean score on MDAS item 5, assessing fear of local anesthetic injection, was 2.1 ± 1.2. Among all respondents, 26.8% scored ≥3, indicating at least moderate injection-related anxiety; 13.7% scored ≥4, and 6.1% scored 5, reflecting high and extreme levels of anxiety.

The age distribution of mean MDAS 5 scores is illustrated in Figure 2. The prevalence of elevated MDAS scores varied by age group. Lower prevalence was observed in older age groups.

**Figure 2:**
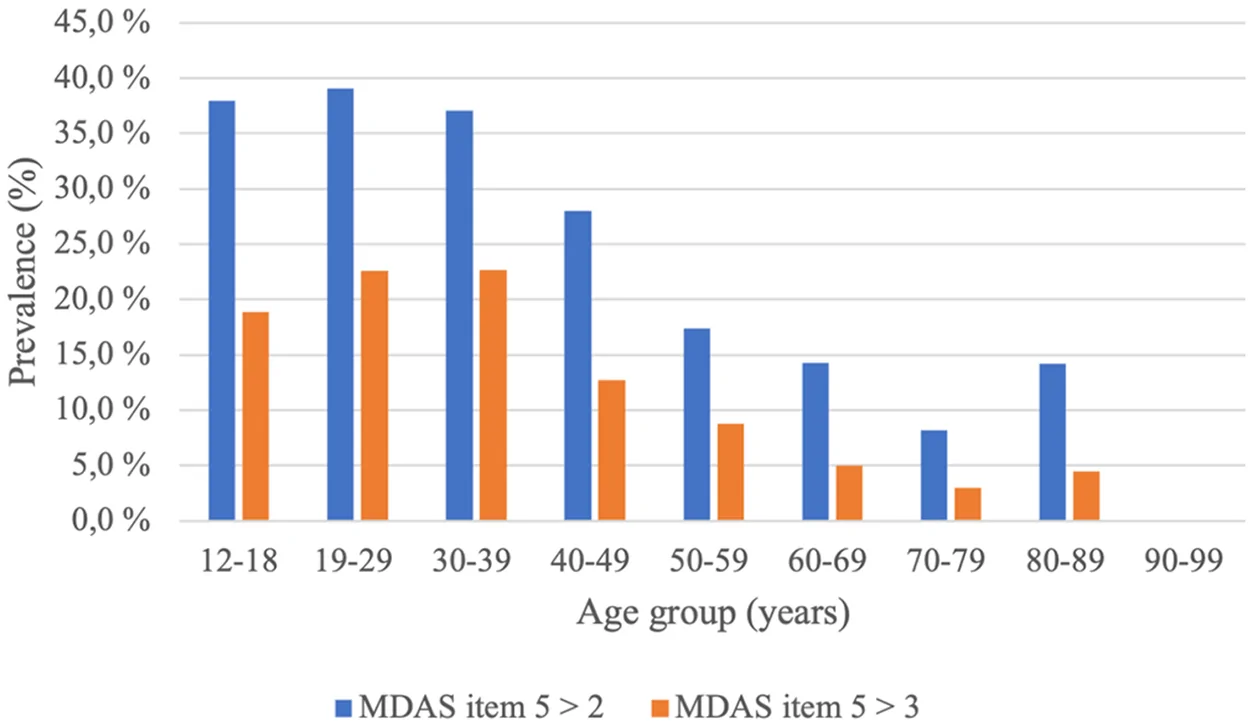
Illustrates two levels of MDAS 5 in different age groups.

The associations between anticipatory anxiety (MDAS items 1–2) and injection-related anxiety was analysed using Pearsons's correlation and significant association was found was positive with correlation 0.59 (*p* > 001).

As shown in Table 1, among participants with low anticipatory anxiety (MDAS 1 + 2 < 6), 20.4% scored >2 on MDAS item 5. In contrast, 80.2% of those with high anticipatory anxiety (MDAS1 + 2 > 6) scored >2, indicating a pronounced gradient in injection-related fear across anticipatory-anxiety categories.

**Table 1:** The association between different levels of anticipatory anxiety **(**MDAS 1 + 2) and anxiety for injections (MDAS 5).

Anticipatory anxiety MDAS 1 + 2	MDAS 5 Fairly anxious Score = 3		MDAS 5 very anxious Score = 4		MDAS 5 Extremely anxious Score = 5		MDAS 5 Score ≥ 3	
	n	%	n	%	n	%	n	%
Not/ slightly anxious Score <6 *N* = 5,208	646	12.4	282	5.4	136	2.6	1,064	20.4
Fairly anxious MDAS = 6 *N* = 263	76	28.9	66	25.1	42	16.0	184	69.9
Very/ extremely anxious MDAS > 6 *N* = 409	49	12.0	97	23.7	182	44.5	328	80.2
Total *N* = 5,880	771	13.1	445	6,1	360	6.1	1,576	26.8

## Discussion

The main findings of this study indicate that general dental practitioners (GDPs) in Norway encounter a substantial proportion of patients with dental anxiety. Approximately one in ten patients presenting for dental care reported moderate dental anxiety (MDAS ≥ 15), while one in twenty met the threshold for high dental anxiety (MDAS ≥ 19). Among patients with high anticipatory anxiety, eight out of ten also reported clinically relevant fear of injections, highlighting the close relationship between these dimensions of dental anxiety.

This study has several strengths. First, the large number of patients included provides a robust basis for estimating the extent of dental anxiety in general dental practice. Second, the sample include patients from across Norway, encompassing both rural and urban areas, and covers a broad age range. Given the known consequences of failing to identify and manage dental anxiety, the high proportion of anxious patients observed in this study underscores the importance of integrating both the diagnosis and management of anxiety into best-practice dental care.

Several limitations should also be considered. The response rate is unknown, as the number of patients who declined participation was not recorded. In addition, the GDPs who recruited participants were not randomly selected; they had enrolled in a course on “special challenges,” including dental anxiety, which may have introduced selection bias by attracting clinicians with a particular interest in the topic and, potentially, a higher proportion of anxious patients. Data collection was also limited to one week, which may not fully represent each dentist's typical patient population. Finally, limited background information was collected, and no data were available on socioeconomic status or general health, which restricts the ability to adjust for potential confounders.

These factors should be considered when interpreting the findings.

The first hypothesis “Dental practitioners (GDPs) in Norway encounter a substantial proportion of patients with dental anxiety” was proven true.

The proportion of patients reporting dental anxiety in this study aligns with previous epidemiological research from Norway (29, 30). Internationally, prevalence estimates vary considerably. Recent studies have reported 7.8% high dental anxiety in Croatia (31) and 8.2% in Greece (32), whereas a study from Lebanon found a markedly higher prevalence of 22.4% dental phobia (33). Such variation likely reflects methodological differences, cultural factors, and heterogeneity in measurement tools.

As the present findings confirm that dental anxiety remains a common condition in clinical dental practice and given its well-documented consequences for oral health across the life course, it appears reasonable to consider systematic identification and management of dental anxiety as an integral component of high-quality general dental care.

Consistent with earlier research, women reported higher levels of dental anxiety in the present study. Twice as many women scored above threshold for moderate dental anxiety (MDAS ≥ 15). This pattern mirrors previous Norwegian findings, where girls scored higher than boys on the Dental Fear Scale (34), and is in line with studies using the MDAS, which have consistently shown higher scores among women across age groups (27, 35). A review of specific phobias further demonstrated that women exhibit higher prevalence rates than men across all included studies, with no clear explanation for the consistently lower prevalence among men (36).

Differences in reporting between women and men may reflect a combination of social and biological factors. Greater social acceptance of fear expression among women, potential biological vulnerabilities, and possible underreporting of anxiety among men, may all contribute to higher anxiety scores in female participants. Gender-specific coping strategies may further influence these patterns; a review by Fisher et al. (37) reported that men were less likely to seek help due to fear of stigma and more likely to rely on self-directed coping or confrontation of feared stimuli directly, rather than avoid them. These mechanisms may help explain why women consistently report higher levels of injection-related anxiety.

A clear age-related pattern was also observed in the present study. For both MDAS ≥ 15 and MDAS ≥ 19, the proportion of participants reporting elevated dental anxiety increased across the three youngest age groups (12–19, 20–29, and 30–39 years). This pattern is consistent with findings reported by Eaton et al. (36), who noted that the incidence of specific phobias tends to peak in adulthood, although the developmental trajectory often begins in childhood. The present results also align with data from Sweden, where the highest levels of dental anxiety were found among individuals aged 20–39 years (38).

Several contextual factors may contribute to this age-related distribution. In Norway, individuals aged 20–39 years no longer have access to free dental care through the Public Dental Service and consequently may not receive regular preventive follow-up. Financial considerations may lead to postponement or avoidance of dental treatment, which in turn can exacerbate dental anxiety over time (2). This mechanism may help explain why anxiety levels appear elevated in this age segment, despite the expectation that dental fear often declines with increasing age.

A central clinical implication of the present findings concerns the role of injection-related anxiety. In the current study, approximately 27% of patients reported being quite anxious or more about injections, 14% reported very high anxiety, and 6% reported extreme anxiety. These figures are consistent with findings from Armfield et al. (39), who reported that around one quarter of an Australian population sample experienced fear of injections.

Age-related patterns were also evident in the reporting of injection-related anxiety. Younger adults expressed higher levels of fear than older age groups, with the highest prevalence observed among individuals aged 19–29 years, where four in ten reported substantial anxiety. Comparable trends have been documented in studies of general injection phobia (40, 41). It is important to note that these studies address general injection phobia rather than fear of oral injections specifically. Given the limited number of studies focusing on dental anesthetic injections, further research is warranted to clarify age-related patterns and to identify potential targets for clinical intervention.

The second hypothesis “patients who report high dental anxiety the day before and in the waiting room before dental treatment have high risk of being anxious for injections of local anesthetics” was found true.

From a clinical perspective, an important observation in this study is the strong link between high anticipatory anxiety and injection-related fear. Among those who were highly anxious the day before treatment or while waiting in the clinic, eight out of ten also reported significant fear of injections. Compared to one of five patients with low anticipatory dental anxiety.

This is highly relevant, as clinical experience indicates that anxious patients who state “let's try without” when they are recommended local anesthesia are not uncommon. Avoidance in this context may be a coping strategy to avoid anxiety. If the GDP is unaware of this risk of an anxiety-driven refusal of injections, consequences of this unfortunate coping strategy is often a painful dental treatment that sustains or exacerbates dental anxiety. The observed positive association between anticipatory dental anxiety and anxiety for intraoral injections, interpreted in light of theories of anxiety and avoidance, highlights that patients with injection-related fear are in risk of postpone or decline local anesthesia if their anxiety is not adequately addressed. This may increase the likelihood of pain, and then in turn further reinforcing dental anxiety. Anticipatory anxiety, loss of control, and previous negative experiences are central mechanisms underlying this pattern.

Early identification of anxious patients, through routine inquiry and attention to verbal and non-verbal cues, is essential to prevent development of this vicious cycle of anticipatory and injection-related anxiety- and consequently further development into Berggrens original vicious circle. Focus on anticipatory dental anxiety enable clinicians to plan treatment in ways that minimize negative experiences and reduce long-term anxiety and poor oral health.

The health ethical principles related to dental care are beneficence, non-maleficence, autonomy, and justice. The dynamics associated with preventing a vicious cycle of avoidance and dental anxiety raise ethical concerns, and GDPs should be aware of their ethical obligation to identify and address injection related fear and anxiety. Yet such issues are seldom discussed in the dental anxiety literature, and current practice suggests that these concerns are often unexplored, leaving many patients without appropriate support or evidence-based management.

First, the dentist is obligated to act beneficially and in accordance with good clinical practice, which includes providing adequate pain control. Second, the duty of non-maleficence implies avoiding painful treatment that is known to increase dental anxiety. These first two principes, however, may conflict with the patients’ autonomy. When a patient refuses local anesthesia, this decision should as a general rule be respected—but the dentist has a responsibility to explore *why* the patient does not want injections, including possible injection-related anxiety or phobia. The final principle, justice, is relevant for addressing a patient's anxiety, but it will require time and resources, which must be balanced against the needs of other patients. These ethical assessments are often challenging in everyday clinical practice and should be assessed on an individually basis, preferably in a systematic and transparent manner.

Both research and clinical experience indicate that adequate pain control is essential for successful management of dental anxiety and helping patients develop a more relaxed relationship with local anesthetic injections is often a critical first step in exposure-based approaches.

In conclusion, this study demonstrates that dental anxiety is prevalent among patients attending general dental practice in Norway. Patients who report anxiety prior to treatment are substantially more likely to fear injections, a combination that can hinder effective pain management and raises important ethical considerations for clinical practice. Given the potential consequences for oral health, the findings underscore the need for increased clinical attention to the early identification and management of dental anxiety. To ensure high-quality care, dentists should proactively assess both anticipatory anxiety and anxiety related to local anesthesia, make case-specific ethical evaluations, and implement appropriate intervention.

## Data Availability

The raw data supporting the conclusions of this article will be made available by the authors, without undue reservation.
